# Dabrafenib and Trametinib in BRAF Mutant Metastatic Conjunctival Melanoma

**DOI:** 10.3389/fonc.2019.00232

**Published:** 2019-04-05

**Authors:** Ernesto Rossi, Brigida Anna Maiorano, Monica Maria Pagliara, Maria Grazia Sammarco, Tommaso Dosa, Maurizio Martini, Guido Rindi, Emilio Bria, Maria Antonietta Blasi, Giampaolo Tortora, Giovanni Schinzari

**Affiliations:** ^1^Medical Oncology, Fondazione Policlinico Universitario “A. Gemelli” IRCCS, Rome, Italy; ^2^Ophtalmology, Fondazione Policlinico Universitario “A. Gemelli” IRCCS, Rome, Italy; ^3^Pathology, Fondazione Policlinico Universitario “A. Gemelli” IRCCS, Rome, Italy

**Keywords:** BRAF, MEK, target therapy, conjunctival, melanoma

## Abstract

Conjunctival melanoma is a rare primary ocular tumor. So far, no standard treatment exists for metastatic disease. Similarly to cutaneous melanoma, up to 50% of conjunctival melanomas harbor BRAF mutations. The most common is represented by V600E. Combined therapy with BRAF and MEK inhibitors is approved for BRAF mutant cutaneous metastatic melanomas. Herein, we report a case of a 70-years old patient with a metastatic conjunctival melanoma harboring V600E BRAF mutation successfully treated with dabrafenib and trametinib.

## Introduction

Conjunctival malignant melanoma is a rare primary ocular tumor with an incidence of 0.2–0.7 per million in Europe and the US (1, 2). Metastases in conjunctival melanoma spread through lymphatic and hematic way, reaching lungs, liver, skin, and brain (3–5). Ten-year mortality rate of conjunctival melanoma is ~30% (5–7). So far, no standard treatment is approved for metastatic conjunctival melanoma.

The Ras/Raf/Mitogen-activated protein kinase (MAPK) pathway has been described as one of the most important regulatory pathways involved in melanoma development (8, 9). MAPK is a signaling cascade starting from receptor-linked tyrosine kinases that activate Ras, then B-raf, MEK1 and MEK2 and finally ERK, that regulates transcriptional factors involved in proliferation, differentiation, apoptosis, and senescence. Around 40–50% of cutaneous melanomas harbor BRAF mutations, that appear at an early stage of tumor development. In fact, activating BRAF mutations have a pro-oncogenic effect. The most common mutation is represented by the substitution of a valine with glutamic acid in the codon 600 (V600E), that increases the catalytic activity of B-raf (10, 11).

Conjunctival melanoma has not been completely characterized at the level of genetics, however, BRAF mutations have been reported in up to 50%, with V600E as the most frequent one (around 80–90%), followed by V600K (2, 8, 12–15).

Treatment with the BRAF inhibitor dabrafenib and the MEK inhibitor trametinib is approved for V600-BRAF mutant patients with cutaneous but not conjunctival melanomas (16–18). In fact, due to its rarity, conjunctival melanoma patients are not included in most clinical trials with BRAF or MEK inhibitors.

Herein, we present a case of a patient with metastatic conjunctival melanoma harboring BRAF V600E mutation who was effectively treated with dabrafenib and trametinib. To the best of our knowledge, there is no previously described treatment response to dabrafenib and trametinib in metastatic conjunctival melanoma.

## Case Description

**Figure 1:**
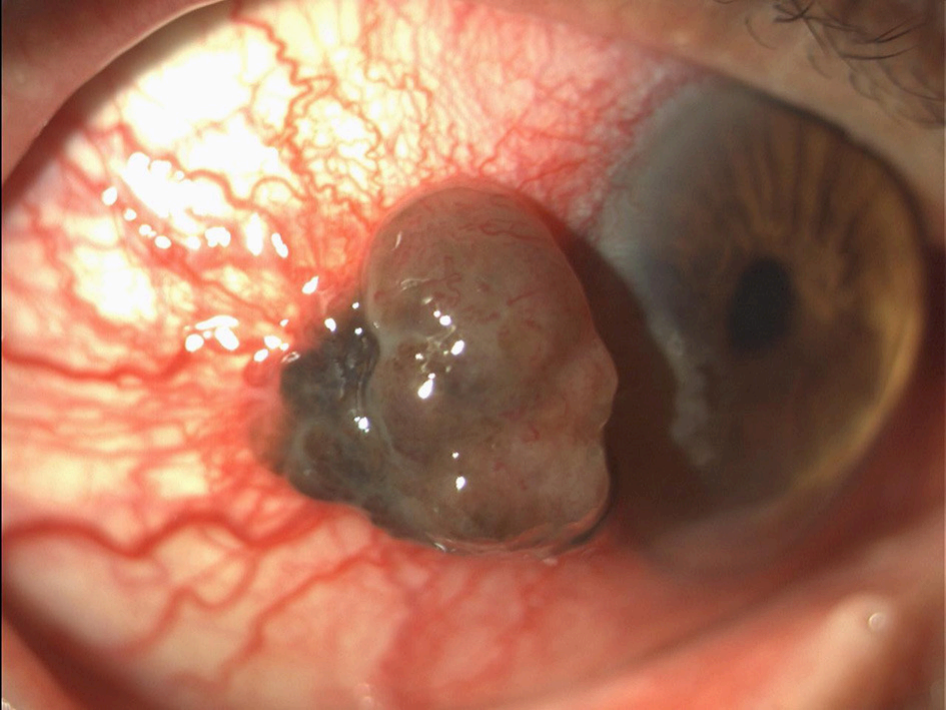
Conjunctival melanoma at presentation.

A 70-years-old Caucasian man presented in April 2016 with a pigmented lesion of about 1 cm located in the temporal limbus of the left bulbar conjunctiva (Figure 1). Patient was in good clinical conditions; the Eastern Cooperative Oncology Group Performance Status (ECOG PS) was 0. No relevant comorbidities or concomitant medications existed. The patient was an officer employee and had no familiarity for neoplasms nor environmental risk factor exposure. The patient underwent a complete escissional biopsy of the bulbar conjunctiva. The histological analysis demonstrated a conjunctival melanoma with a thickness of 0.3 cm. The extracted DNA was amplified with specific primers for exon 15 of BRAF gene and then sequenced. Codon 600 was also analyzed through Idylla™ BRAF mutation test. The heterozygote mutation of codon V600 was detected, but the specific amminoacidic substitution was not identified due to the scarcity of histological tissue.

In July 2017, the patient noticed a tumefaction of the right parotid region. The ^18^fluoro-desossi-glucose (FDG) positron emission tomography (PET)/computerized tomography (CT) scan showed an uptake in a nodule of the right parotid gland (ø 23 mm) and latero-cervical lymph node metastases with a maximal standard uptake value (SUV) of 4.4. The fine needle aspiration cytology (FNAC) revealed melanoma cells. Therefore, the patient underwent a right parotidectomy with latero-cervical lymph node dissection. The histologic exam confirmed the parotid metastasis from conjunctival melanoma. Metastasis of melanoma was also detected in 1/13 lymph nodes. There was no evidence of extra nodal invasion. The extracted DNA was amplified with specific primers for exon 15 of BRAF gene and then sequenced. V600E mutation (T1799A) was detected.

**Figure 2:**
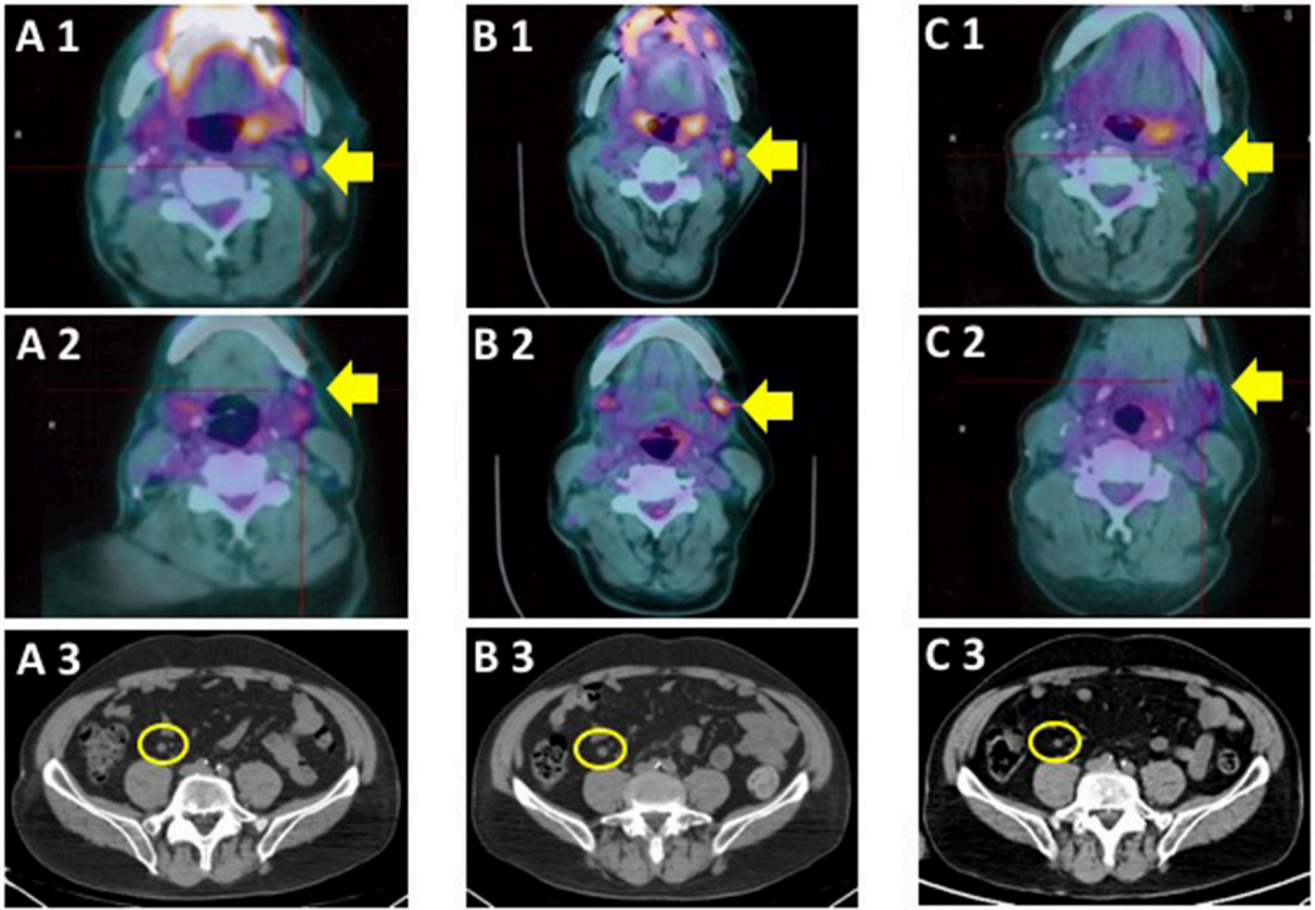
Comparison of ^18^FDG-PET/CT scan at baseline **(A)**, first **(B)** and second **(C)** assessment during the treatment. 1: left retro-mandibular lymph node, indicated by yellow arrows; 2: left latero-cervical lymph node, indicated by yellow arrows; 3: nodule in the mesenteric adipose tissue in right hypogastrium, circled.

The ^18^FDG PET scan after intervention (October 2017), revealed a residual tumor in the lymph nodes located in left retro-mandibular and latero-cervical areas (maximal SUV 3.9) and a nodule in the mesenteric adipose tissue in right hypogastrium (Figure 2A). LDH was 700 IU/L (ULN 450 IU/L). Other cases of BRAF mutant conjunctival melanoma experienced a disease control with BRAF inhibition therapy (Table 1). In metastatic cutaneous melanoma, the combination of anti-BRAF and anti-MEK demonstrated better survival results vs. anti-BRAF alone. Indeed, we hypothesized that the target combination therapy could be the best option also for this BRAF mutant conjunctival melanoma patient. The combination of these drugs is not approved for conjunctival melanoma, thus we requested the off-label use in accordance with our institutional policy. After the institutional permission, the written consent was obtained by the patient. Then we started therapy with Dabrafenib (150 mg twice daily) and Trametinib (2 mg daily), choosing the standard dosage approved for metastatic cutaneous melanoma (24).

**Table 1:** Previous reports of treatment with BRAF and/or MEK inhibitors in conjunctival melanoma patients.

**References**	**Disease sites**	**Drug**	**Duration**	**Side effects**	**Outcome**
Pahlitzsch et al. (19)	Local disease	Vemurafenib (not specified dose)	16 months	Weight loss, nausea and vomiting, headaches	Local control
Pinto Torres et al. (20)	Local recurrent disease	Vemurafenib (960 mg twice a day, reduced to 480 mg twice a day due to toxicity)	34 months	Grade 2 diarrhea, grade 2 arthralgia, grade 1 skin rash, needing a dose reduction	Complete response
Maleka et al. (21)	Pre-treated metastatic disease	Vemurafenib (960 mg twice a day, reduced to 720 mg twice a day due to toxicity)	4 months	Grade 2 maculopapular rash, needing a dose reduction	Partial response
Griewank et al. (22)	Pre-treated metastatic disease	Dabrafenib (150 mg twice a day)	6 months	[Not reported]	Partial response
Dagi Glass et al. (23)	Locally advanced disease	Dabrafenib and Trametinib interrupted for toxicity	1.5 months	Nausea and vomiting, leading to therapy discontinuation	Patient followed for over 23 mo without distant spread
		Vemurafenib	3.5 months	[Not reported]	
		Pembrolizumab	2 months		
		Vemurafenib and Cobimetinib (added after 4 months).	Not reported	[Not reported]	

Vital signs, dermatological exam and biochemistry analysis had been performed monthly. The therapy was complicated by fever with body temperature between 37.5 and 38°C. No signs of infections were detected. Patient took paracetamol for the fever and the adverse event resolved without squeal after 2 months. No dose reduction or interruption was needed.

The first ^18^FDG-PET/CT scan after 4 months of therapy revealed the persistence of lymph node disease (with increased maximal SUV to 4.4), while the mesenteric nodule was stable (Figure 2B). The cranial CT scan with contrast enhancement did not reveal any sign of tumor recurrence. The combination of Dabrabenib/Trametinib was continued.

The following ^18^FDG-PET/CT scan was performed after further 4 months of therapy. It showed the reduction of the metabolic activity of the lymph node metastases of the neck (maximal SUV 3). The mesenteric nodule was not modified (Figure 2C). The magnetic resonance imaging (MRI) of the brain did not reveal ocular recurrence of melanoma nor encephalic metastases.

Ophthalmic examinations have been performed every 6 months, and they detected no signs of uveitis or iridocyclitis. The patient never complained of photophobia, ocular pain, or other visual alterations.

The blood analysis performed in August 2018 revealed a grade 1 hypertransaminasemia.

On September 2018, the treatment with dabrafenib and trametinib is ongoing at the initial dose. No dose reduction was necessary.

## Discussion

Over the last years, therapy for metastatic cutaneous melanoma has been strengthened by target therapies, including treatments against the MAPK pathway. Dabrafenib is a highly selective inhibitor of mutated BRAF and trametinib inhibits MEK1 and MEK2. The combination of BRAF and MEK inhibitors shows response rates up to 70% with an improved survival in metastatic cutaneous melanoma harboring BRAF mutations of the codon 600 (16–18, 25). NCT01584648 registration trial showed a median progression free survival for V600-BRAF mutant melanoma patients treated with dabrafenib plus trametinib of 9.3 months, and an overall survival of 25 months (17, 24).

It has been shown that activating BRAF mutations are detectable in up to 50% of conjunctival melanomas, of which about 80% are V600E, 15–20% V600K (15, 26). *In vitro*, Vemurafenib and Dabrafenib inhibit the growth of BRAF-mutated conjunctival melanoma cell lines; MEK inhibitors also promote apoptosis of cell lines from conjunctival melanoma (27). However, neither BRAF inhibitors nor MEK inhibitors are yet approved for patients with conjunctival melanomas.

Previous attempts of treating metastatic conjunctival melanomas with BRAF inhibitors have been reported (Table 1). A patient with a BRAF-mutant conjunctival melanoma that refused the exenteration of the orbit was treated with BRAF inhibitor Vemurafenib for 16 months, obtaining a local control that permitted a more conservative surgery. The patient developed weight loss, nausea, and vomiting (19).

A second patient with a recurrent BRAF mutant conjunctival melanoma was effectively treated with Vemurafenib, experiencing a complete remission for over 3 years. Dose reduction was needed because of toxicity, represented by grade 2 diarrhea, grade 2 arthralgia, and grade 1 skin rash (20).

A third patient with a BRAF mutant pre-treated metastatic conjunctival melanoma benefitted from Vemurafenib with a partial response for about 4 months. Grade 2 maculopapular rash was reported, needing a dose reduction (21).

Another patient with pre-treated metastatic conjunctival melanoma expressing BRAF mutation developed a partial response for over 6 months of therapy with the BRAF inhibitor Dabrafenib (22).

Another report described a patient with a locally advanced conjunctival melanoma that was initially treated with combined BRAF/MEK inhibitors (Dabrafenib and Trametinib). After 1.5 months, the treatment was interrupted for toxicity represented by nausea and vomiting, and Vemurafenib alone was continued. Therapy was interrupted 3.5 months later for progressive disease to start Pembrolizumab. After progression, firstly the BRAF inhibitor alone (Vemurafenib) was restarted and then a MEK inhibitor (Cobimetinib) was added. The patient was followed for over 23 months without evidence of distant spread (23).

In advanced cutaneous melanoma, the combined inhibition of BRAF/MEK in case of BRAF mutation is effective. The lack of clinical trial does not allow to consider this mutation as a therapeutic target in conjunctival melanoma. However, the patient described in this case report achieved a clinical benefit from this treatment. Thus, it is conceivable that patients with BRAF-mutant conjunctival melanoma could benefit from treatment with BRAF/MEK inhibitors.

## Conclusions

No standard treatment for metastatic conjunctival melanoma has been established. To our knowledge, we present the first patient with a BRAF-mutant metastatic conjunctival melanoma who has been effectively treated with a combined use of BRAF/MEK inhibitor. BRAF mutations need to be investigated in conjunctival melanomas. Further studies are needed to assess the efficacy of BRAF and MEK inhibitors in this subtype of ocular melanoma.

## Ethics Statement

Written informed consent, according to the Declaration of Helsinki, for treatment and publication of this case report, including the images, was obtained from the patient.

## Author Contributions

All authors listed have made a substantial, direct and intellectual contribution to the work, and approved it for publication.

### Conflict of Interest Statement

The authors declare that the research was conducted in the absence of any commercial or financial relationships that could be construed as a potential conflict of interest.

## References

[1] McLaughlin CC Wu XC Jemal A Martin HJ Roche LM Chen VW Incidence of non-cutaneous melanomas in the US. Cancer. (2005) 103:1000–7. 10.1002/cncr.20866 15651058

[2] Kastelan S Gverović Antunica A Orešković LB Rabatic JS Kasun B Bakija I. Conjunctival melanoma - epidemiological trends and features. Pathol Oncol Res. (2018) 24:787–96. 10.1007/s12253-018-0419-3 29802540

[3] Shields CL Shields JA Gündüz K Cater J Mercado GV Gross N. Conjunctival melanoma: risk factors for recurrence, exenteration, metastasis, and death in 150 consecutive patients. Arch Ophthalmol. (2000) 118:1497–507. 10.1001/archopht.118.11.1497 11074806

[4] Esmaeli B Wang X Youssef A Gershenwald JE. Patterns of regional and distant metastasis in patients with conjunctival melanoma: experience at a cancer over four decades. Ophthalmol. (2001) 108:2101–5. 10.1016/S0161-6420(01)00782-5 11713086

[5] Missotten GS Keijser S De Keizer RJ De Wolff-Rouendaal D. Conjunctival melanoma in the Netherlands: a nationwide study. Invest Ophthalmol Vis Sci. (2005) 46:75–82. 10.1167/iovs.04-0344 15623757

[6] Triay E Bergman L Nilsson B All-Ericsson C Seregard S. Time trends in the incidence of conjunctival melanoma in Sweden. Br J Ophthalmol. (2009) 93:1524–8. 10.1136/bjo.2009.157933 19628487

[7] Shildkrot Y Wilson MW. Conjunctival melanoma: pitfalls and dilemmas in management. Curr Opin Ophthalmol. (2010) 21:380–6. 10.1097/ICU.0b013e32833b7aab 20531189

[8] Spendlove HE Damato BE Humphreys J Barker KT Hiscott PS Houlston RS BRAF mutations are detectable in conjunctival but not uveal melanomas. Melanoma Res. (2004) 14:449–52. 10.1097/00008390-200412000-00003 15577314

[9] Dahl C Guldberg P. The genome and epigenome of malignant melanoma. APMIS. (2007) 115:1161–76. 10.1111/j.1600-0463.2007.apm_855.xml.x 18042149

[10] Goel VK Lazar AJ Warneke CL Redston MS Haluska FG. Examination of mutations in BRAF, NRAS, and PTEN in primary cutaneous melanoma. J Invest Dermatol. (2006) 126:154–60. 10.1038/sj.jid.5700026 16417231

[11] Omholt K Platz A Kanter L Ringborg U Hansson J. NRAS and BRAF mutations arise early during melanoma pathogenesis and are preserved throughout tumor progression. Clin Cancer Res. (2003) 9:6483–8. 14695152

[12] Seregard S. Conjunctival melanoma. Surv Ophthalmol. (1998) 42:321–50. 9493274 10.1016/s0039-6257(97)00122-7

[13] Gear H Williams H Kemp EG Roberts F. BRAF mutations in conjunctival melanoma. Invest Ophthalmol Vis Sci. (2004) 45:2484–8. 10.1167/iovs.04-0093 15277467

[14] Lake SL Jmor F Dopierala J Taktak AF Coupland SE Damato BE. Multiplex ligation-dependent probe amplification of conjunctival melanoma reveals common BRAF V600E gene mutation and gene copy number changes. Invest Ophthalmol Vis Sci. (2011) 52:5598–604. 10.1167/iovs.10-6934 21693616

[15] Griewank KG Westekemper H Murali R Mach M Schilling B Wiesner T. Conjunctival melanomas harbor BRAF and NRAS mutations and copy number changes similar to cutaneous and mucosal melanomas. Clin Cancer Res. (2013) 19:3143–52. 10.1158/1078-0432.CCR-13-0163 23633454

[16] Flaherty KT Infante JR Daud A Gonzalez R Kefford RF Sosman J. Combined BRAF and MEK inhibition in melanoma with BRAF V600 mutations. N Engl J Med. (2012) 367:1694–703. 10.1056/NEJMoa1210093 23020132 PMC3549295

[17] Long GV Stroyakovskiy D Gogas H Levchenko E de Braud F Larkin J. Dabrafenib and trametinib versus dabrafenib and placebo for Val600 BRAF-mutant melanoma: a multicentre, double-blind, phase 3 randomised controlled trial. Lancet. (2015) 386:444–51. 10.1016/S0140-6736(15)60898-4 26037941

[18] Grob JJ Amonkar MM Karaszewska B Schachter J Dummer R Mackiewicz A. Comparison of dabrafenib and trametinib combination therapy with vemurafenib monotherapy on health-related quality of life in patients with unresectable or metastatic cutaneous BRAF Val600-mutation-positive melanoma (COMBI-v): results of a phase 3, open-label, randomised trial. Lancet Oncol. (2015) 16:1389–98. 10.1016/S1470-2045(15)00087-X 26433819

[19] Pahlitzsch M Bertelmann E Mai C Conjunctival melanoma and BRAF inhibitor therapy. J Clin Exp Ophthalmol. (2014) 5:322 10.4172/2155-9570.1000322

[20] Pinto Torres S André T Gouveia E Costa L Passos MJ. Systemic treatment of metastatic conjunctival melanoma. Case Rep Oncol Med. (2017) 2017:4623964. 10.1155/2017/4623964 29214089 PMC5682064

[21] Maleka A Astrom G Byström P Ullenhag GJ. A case report of a patient with metastatic ocular melanoma who experienced a response to treatment with the BRAF inhibitor vemurafenib. BMC Cancer. (2016) 16:634. 10.1186/s12885-016-2657-7 27520988 PMC4983009

[22] Griewank KG Westekemper H Schilling B Livingstone E Schimming T Sucker A. Conjunctival melanomas harbor BRAF and NRAS mutations–letter. Clin Cancer Res. (2013) 19:6331–2. 10.1158/1078-0432.CCR-13-2368 24170549

[23] Dagi Glass LR Lawrence DP Jakobiec FA Freitag SK. Conjunctival melanoma responsive to combined systemic BRAF/MEK inhibitors. Ophthalmic Plast Reconstr Surg. (2017) 33:e114–6. 10.1097/IOP.0000000000000833 27893585

[24] Long GV Stroyakovskiy D Gogas H Levchenko E de Braud F Larkin J. Combined BRAF and MEK inhibition versus BRAF inhibition alone in melanoma. N Engl J Med. (2014) 371:1877–88. 10.1056/NEJMoa1406037 25265492

[25] Robert C Karaszewska B Schachter J Rutkowski P Mackiewicz A Stroiakovski D. Improved overall survival in melanoma with combined dabrafenib and trametinib. N Engl J Med. (2015) 372:30–9. 10.1056/NEJMoa1412690 25399551

[26] Larsen AC Dahmcke CM Dahl C Siersma VD Toft PB Coupland SE. A retrospective review of conjunctival melanoma presentation, treatment, and outcome and an investigation of features associated with BRAF mutations. JAMA Ophthalmol. (2015) 133:1295–303. 10.1001/jamaophthalmol.2015.3200 26425792

[27] Cao J Heijkants RC Jochemsen AG Dogrusöz M de Braud MJ van der Velden PA. Targeting of the MAPK and AKT pathways in conjunctival melanoma shows potential synergy. Oncotarget. (2016) 8:58021–36. 10.18632/oncotarget.10770 28938534 PMC5601630

